# Cotargeting of XPO1 Enhances the Antileukemic Activity of Midostaurin and Gilteritinib in Acute Myeloid Leukemia

**DOI:** 10.3390/cancers12061574

**Published:** 2020-06-14

**Authors:** Lindsey T. Brinton, Steven Sher, Katie Williams, Daniel Canfield, Shelley Orwick, Ronni Wasmuth, Casey Cempre, Jordan Skinner, Amy Lehman, James S. Blachly, John C. Byrd, Rosa Lapalombella

**Affiliations:** 1 Division of Hematology, Department of Internal Medicine, The Ohio State University, Columbus, OH 43210, USA; Lindsey.Brinton@osumc.edu (L.T.B.); Steven.Sher@osumc.edu (S.S.); Katie.Williams@osumc.edu (K.W.); Daniel.Canfield@osumc.edu (D.C.); Shelley.Orwick@osumc.edu (S.O.); Ronni.Wasmuth@osumc.edu (R.W.); Casey.Cempre@osumc.edu (C.C.); Jordan.Skinner@osumc.edu (J.S.); James.Blachly@osumc.edu (J.S.B.); John.Byrd@osumc.edu (J.C.B.); 2 Center for Biostatistics, The Ohio State University, Columbus, OH 43210, USA; Amy.Lehman@osumc.edu; 3 Leukemia Research Program, The Ohio State University James Comprehensive Cancer Center, Columbus, OH 43210, USA; 4 Department of Biomedical Informatics, The Ohio State University, Columbus, OH 43210, USA; 5 College of Pharmacy, The Ohio State University, Columbus, OH 43210, USA; 6 College of Veterinary Medicine, The Ohio State University, Columbus, OH 43210, USA

**Keywords:** AML, FLT3, XPO1, CRISPR-Cas9 screening, synergism

## Abstract

Acute myeloid leukemia (AML) is a hematopoietic stem-cell-derived leukemia with often successive derived driver mutations. Late onset acquisition of internal tandem duplication in *FLT3* (*FLT3*-ITD) at a high variant allele frequency often contributes to full transformation to a highly proliferative, rapidly progressive disease with poor outcome. The *FLT3*-ITD mutation is targetable with approved FLT3 small molecule inhibitors, including midostaurin and gilteritinib. However, outside of patients receiving allogeneic transplant, most patients fail to respond or relapse, suggesting alternative approaches of therapy will be required. We employed genome-wide pooled CRISPR knockout screening as a method for large-scale identification of targets whose knockout produces a phenotypic effect that enhances the antitumor properties of FLT3 inhibitors. Among the candidate targets we identified the effect of XPO1 knockout to be synergistic with midostaurin treatment. Next, we validated the genetic finding with pharmacologic combination of the slowly reversible XPO1 inhibitor selinexor with midostaurin and gilteritinib in *FLT3*-ITD AML cell lines and primary patient samples. Lastly, we demonstrated improved survival with either combination therapy compared to its monotherapy components in an aggressive AML murine model, supporting further evaluation and rapid clinical translation of this combination strategy.

## 1. Introduction

Acute myeloid leukemia (AML) results from the accumulation of somatic chromosomal abnormalities, genetic mutations, and epigenetic changes that disrupt normal hematopoietic maturation and allow for expansion of undifferentiated myeloid precursor cells. Indeed, the pathogenesis of AML often involves one or more cytogenetic abnormalities that blocks hematopoietic differentiation combined with mutations in either pro-apoptotic genes, such as *TP53*, or proliferative kinase genes, such as *FLT3*, *c-Kit*, and *RAS*-related family members. Acquisition of activated kinase genes often leads to rapidly proliferative disease. Despite being the most incident adult leukemia, AML clinical outcome with a common intensive chemotherapy approach not directed toward the specific driving cytogenetic or mutational feature of AML has been relatively poor due largely to inter- and intrapatient heterogeneity of the disease [1].

Approximately 30% of patients with acute myeloid leukemia (AML) have an internal tandem duplication (ITD) or tyrosine kinase domain (TKD)-activating mutation in the *fms-like tyrosine kinase 3 gene* (*FLT3*). The *FLT3*-ITD mutation is by far the most common of the two common mutations of FLT3, and when present at a high variant allele frequency (often measured as high >0.5 *FLT3*-ITD allelic ratio) in the entire leukemia clonal population, it promotes a more aggressive disease phenotype and poor prognosis [2,3]. Indeed, the European Leukemia Net (ELN) categorizes *FLT3*-ITD with a high allelic ratio in the high risk group of patients, whereas a *FLT3*-TKD or a low allelic ratio (<0.5) and absence of other high risk features results in intermediate classification [1]. The advent of first and second generation FLT3 inhibitors opened a new avenue of targeted treatment for these patients. The first generation FLT3 inhibitor midostaurin and more potent second generation gilteritinib are FDA-approved for marketing in this setting and have been shown to prolong survival of patients with *FLT3*-mutated AML, either in combination with standard chemotherapy as initial treatment (midostaurin) or as salvage therapy (gilteritinib) [4,5]. However, FLT3 inhibitors alone or in combination are insufficient to induce durable remission in the majority of patients [6]. We hypothesized that the antileukemic activity of FLT3 inhibitors could be enhanced by combinatorial therapy with agents targeted against specific gene targets versus classic DNA damaging agents. To uncover genes whose knockout produces a phenotypic effect that synergize with the effect of inhibition of FLT3 in promoting cell death we conducted genome-wide Clustered Regularly Interspaced Short Palindromic Repeats (CRISPR)/Cas9 loss-of-function screens in a human AML cell line with application of selective pressure from midostaurin. We next prioritized targets with clinical grade therapeutics that could immediately be employed for clinical trials. Among these top synergistic hits, we identified *Exportin-1* (*XPO1*).

XPO1 has been recognized as a potential therapeutic target in hematologic and solid tumors. Selective inhibitors of XPO1 have been shown to possess antileukemic and proapoptotic activity in AML blasts by disrupting aberrant removal of tumor suppressor proteins from the nucleus, such as TP53 [7]. XPO1 inhibition was effective preclinically in prolonging survival in murine models bearing *FLT3*-ITD leukemic cells. However, despite some clinical benefit in vivo in a human clinical trial of AML, the anti-XPO1 drug selinexor was not sufficiently active as a monotherapy in relapsed disease versus other salvage agents [8]. Given the known impact of XPO1 inhibition on the self-renewal of leukemia-initiating cells (LICs) and the high expression of XPO1 in *FLT3*-ITD patients [9], combination of selinexor with FLT3 inhibitors could synergistically impact disease burden for *FLT3*-ITD AML patients and further extend the antileukemic benefit to the LIC compartment [10].

Through genetic and pharmacologic validation, we showed that inhibition of XPO1 enhanced the activity of both midostaurin and gilteritinib ex vivo in AML primary patient blasts harboring *FLT3*-ITD, building upon prior work examining selinexor in combination with the tyrosine kinase inhibitor sorafenib, which has anti-FLT3 activity [11]. Furthermore, we demonstrated prolonged survival of the combination therapies selinexor plus midostaurin or selinexor plus gilteritinib over monotherapy alone in an AML xenograft mouse model thus validating the CRISPR screen prediction. Our data suggest this combination strategy enhances the effect of some of the most effective targeted AML drugs available, midostaurin and gilteritinib, and has the potential to translate rapidly to the clinic and induce durable remission in patients with this genomic abnormality.

## 2. Results

### 2.1. Loss-of-Function Screening Reveals the Effect of XPO1 Knockout as Potentially Synergistic with Midostaurin

**Figure 1:**
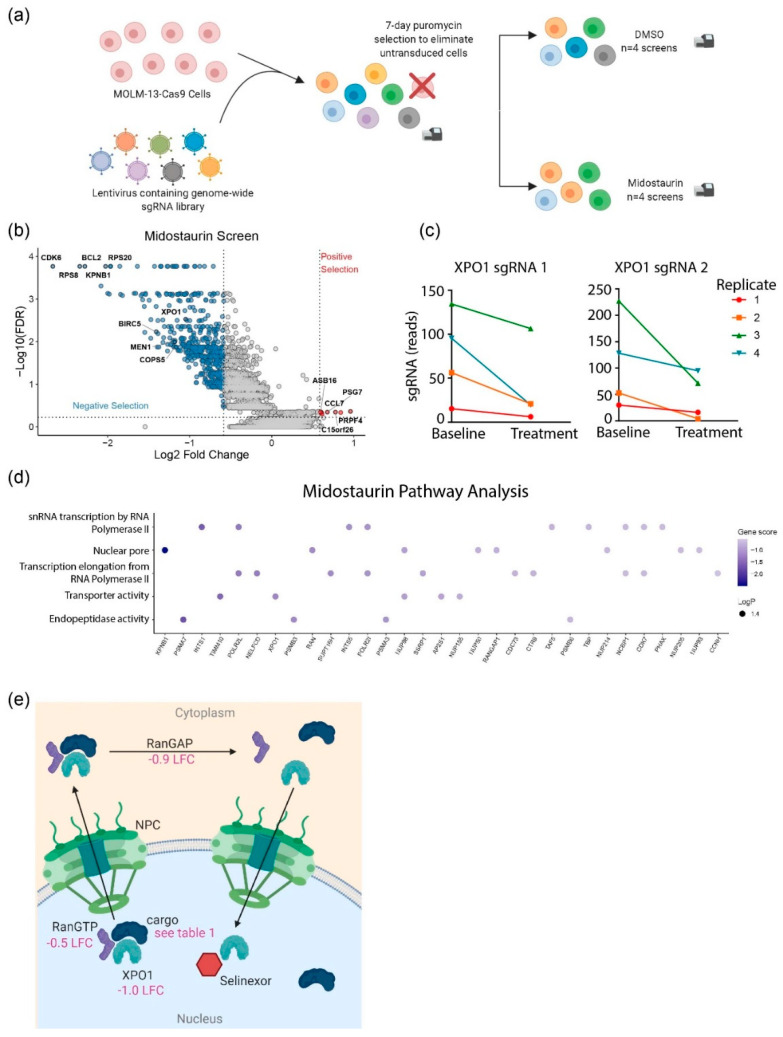
Midostaurin CRISPR knockout screen. (**a**) Screen design. (**b**) Volcano scatter plot showing significant positive and negative selection results. (**c**) Changes in levels of the two most efficient sgRNAs targeting XPO1 in four replicate screens. (**d**) Enriched gene pathway analysis. (**e**) Schematic of nuclear pore complex (NPC) components with log-fold change (LFC) values from the screen indicated.

To identify genes that, when cotargeted, produce a phenotypic effect that can enhance the effect of midostaurin treatment in AML patients, we conducted genome-wide loss-of-function screens with the human CRISPR knockout (KO) pooled library “Brunello” from the Broad Institute (Addgene#73179) [12] applying selective pressure with midostaurin, a multikinase inhibitor with high specificity for mutant FLT3, but not wildtype FLT3. MOLM-13 cells were inoculated at a low multiplicity of infection (MOI) and treated with 30 nM of continuous midostaurin or dimethyl sulfoxide (DMSO 0.00012%) as a vehicle control for five days, with four biological replicates (Figure 1a). CRISPR screens passed all established quality metrics (Figure S1a–c) [13]. Candidate hits were segregated into statistically significant negative selection (synergistic) and positive selection (resistant) hits via a robust ranking algorithm (RRA) [13] (Figure 1b), with the strongest hits demonstrating a consistent pattern among multiple sgRNAs targeting that gene (Figure 1c).

Among the statistically significant synergistic hits for which clinically relevant FDA marketed agent inhibitors are approved, we identified *XPO1* (Table S1) and *BCL2*. The rationale for combination therapy of midostaurin and XPO1 inhibitors was strongly supported by pathway analysis (Figure 1d), which identified—in addition to the nuclear export pathway of which XPO1 is a key protein—the nuclear pore pathway, which includes proteins such as Ran and RanGAP1 that are known to bind with XPO1 [14]. Furthermore, we examined genes coding for known interacting partners of XPO1 from literature [15,16,17,18] and the comprehensive database NESdb [19] and found that the effect of the knockout of many of these genes was also predicted to be synergistic with midostaurin (Figure 1e; Table 1); the depletion was especially strong for Karyopherin-β1 (KPNB1) and Baculoviral IAP Repeat Containing 5 (BIRC5). BCL2 inhibition with venetoclax has been already shown to sensitize *FLT3*-mutated AML cell lines to gilteritinib [20]. A Phase 1b study of venetoclax and gilteritinib in patients with Relapsed/Refractory AML also demonstrated efficacy, with 50% of FLT3-mutant patients achieving composite complete remission and an additional 40% reaching a morphologic leukemia-free state [21]. Preliminary data suggests this combination to be well tolerated and demonstrated blast clearance in 90% of patients with *FLT3*-mutated AML.

Lastly, mapping of synergism and resistance predictions onto known FLT3 signaling cascades in AML [22] highlights the dependence of *FLT3*-ITD AML on enhanced downstream MAPK signaling, and predicts that deactivating Ras mutations would promote synergy; this is in line with recent work showing that activating Ras mutations can promote FLT3 inhibitor resistance clinically (Figure S2) [23]. XPO1 has a known role in MAPK cellular processes, which further suggests that inhibition of XPO1 could act synergistically with midostaurin treatment and may also prevent development of resistance. Together, these analyses provide strong validation of our CRISPR screen results and support further validation of XPO1 KO as a rational combination strategy with midostaurin.

### 2.2. Genetic Knockdown of XPO1 Enhances the Effect of Midostaurin and Gilteritinib in *FLT3*-ITD Cell Lines

**Figure 2:**
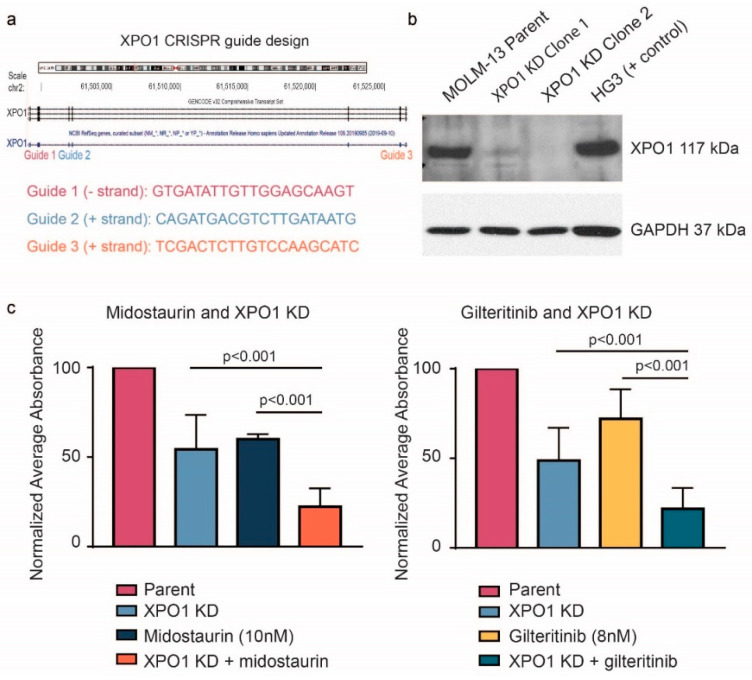
Genetic validation of XPO1 as a cotarget with FLT3. (**a**) Design of three guides targeting XPO1 used to knock down XPO1 expression in MOLM-13 cells. (**b**) Immunoblot analysis showing protein expression of XPO1 in MOLM-13 cells, XPO1 knockdown (KD) cell lines, and HG3 (positive control). (**c**) MOLM-13 parent cells and XPO1 knockdown cells were treated with 10 nM midostaurin or 8 nM gilteritinib for 48 h and proliferation changes compared. For each knockdown experiment, conditions were compared with each clone separately and then the results pooled together across clones to get an average effect (*p* < 0.001 where noted).

Based on the above results, we examined the in vitro relevance of the loss of XPO1 on midostaurin-mediated cell death in *FLT3*-ITD AML cells. We first knocked down (KD) levels of XPO1 protein expression with CRISPR ribonucleoprotein in the same MOLM-13 cell line model of *FLT3*-ITD AML as used in screening with three different guides (Figure 2a) and confirmed reduced protein expression via immunoblot analysis (Figure 2b). Next, we tested if the reduced expression of XPO1 increased the cytotoxic effect of midostaurin treatment as predicted by our earlier described CRISPR screen. As shown in Figure 2c, midostaurin treatment alone decreased viable cells, as did XPO1 KD, but the combination of both the XPO1 KD and midostaurin caused a stronger reduction in viability (*p* < 0.001). Because midostaurin exerts additional non-FLT3 kinase inhibitory activity, we sought to reproduce this effect with the more selective and potent FLT3 inhibitor gilteritinib, which has enhanced in vivo activity and monotherapy activity in patients with AML [24]. Similar to the midostaurin treatment, gilteritinib alone and XPO1 KD alone both decreased viable cells compared to control-treated parental cells, but the combination of gilteritinib and XPO1 KD resulted in a much stronger reduction (*p* < 0.001; Figure 2c). These studies confirm that XPO1 is a viable target for combination therapy with both first and second generation FLT3 inhibitors. These findings justify pursuit of pharmacologic inhibition of XPO1 inhibition together with FLT3 inhibitors to determine if similar synergy is observed.

### 2.3. Pharmacologic Inhibition of XPO1 via Selinexor is HSA Synergistic with FLT3 Inhibition by Either Midostaurin or Gilteritinib in *FLT3*-ITD AML Cell Lines and Primary Patient Samples

**Figure 3:**
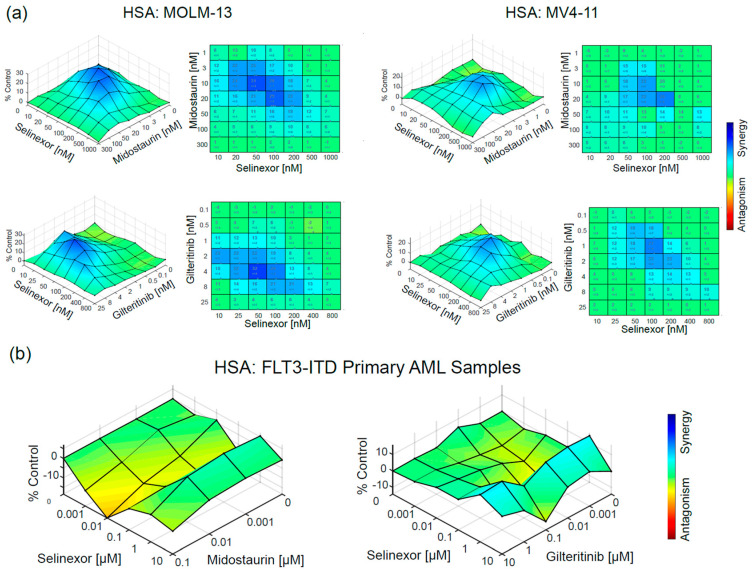
In vitro pharmacologic validation of XPO1 as a cotarget with FLT3. (**a**) MOLM-13 or MV4-11 cells were treated with a range of doses of midostaurin or gilteritinib plus selinexor for 48 h and proliferation changes measured. Regions of synergy were determined using highest single agent analysis shown here; mathematical synergy was calculated in Figure S3. (**b**) Similar proliferation and synergy analysis was applied to primary *FLT3*-ITD AML patient samples, AML1 and AML2, which were cocultured with HS5 stromal cells for 96 h. For each drug (*n* = 2 patient samples), blast cells were separated from stroma prior to development with MTS and results averaged prior to analysis against a highest single agent model shown here; mathematical synergy was calculated in Figure S4.

Selinexor is a slowly reversible inhibitor of XPO1 with proven preclinical activity in a variety of AML tumor cells. Additionally, the use of selinexor has recently received accelerated FDA approval in patients with relapsed multiple myeloma, making it immediately available for clinical combination with a FLT3 inhibitor. We therefore focused on treatment of *FLT3*-ITD AML cell lines with a range of doses of selinexor together with a range of doses of either midostaurin or gilteritinib to determine the synergistic range for the combination. For MOLM-13 cells, the range of synergy was 10–200 nM selinexor with 3–50 nM midostaurin or 2–8 nM gilteritinib (Figure 3a and Figure S3), which matches pharmacologically achievable doses in patients [8,25,26]. Peak synergy on MOLM-13 cells was at 50 nM selinexor with 10 nM midostaurin or 4 nM gilteritinib. Utilizing an alternative *FLT3*-ITD positive AML cell line, MV4-11, we demonstrated that the range of synergy was 20–200 nM selinexor with 3–20 nM midostaurin or 0.5–8 nM gilteritinib. Peak synergy on MV4-11 cells was at 100 nM selinexor with 20 nM midostaurin or 1 nM gilteritinib. Single-agent dose responses indicate that both cell lines are more sensitive to gilteritinib than midostaurin, as expected (Figure S3a,b). The synergistic window is consistent with what could be achieved therapeutically, and prompted us to evaluate the combination therapy in primary patient samples, which are inherently more variable in their response to drugs, have more heterogeneity than cultured cell lines, and are a helpful model for how a drug combination might work in a specific genomic population.

To confirm that our multicell line *FLT3*-ITD AML work was relevant to primary AML samples, we cocultured AML primary patient samples (Figure S4a, *n* = 2) having a high allelic ratio of *FLT3*-ITD with HS5 stroma as a support system to enhance survival for 96 h. Samples were treated with a range of doses of selinexor in combination with either midostaurin or gilteritinib and both demonstrated some level of synergy (Figure 3b and Figure S4b,c).

The combination of midostaurin and selinexor revealed mild synergy commensurate to a minimal dose–response effect to midostaurin; whereas stronger synergy was evident in the combination of gilteritinib and selinexor with a corresponding higher dose response to gilteritinib toward *FLT3*-ITD AML (Figure S4d). The comparatively higher doses indicative of synergy in the primary patient sample proliferation assays versus those in the cell line proliferation assays are also present at the single-agent level (Figure S3a,b). For example, the EC50 of gilteritinib is 3.46 µM and 1.30 µM for AML1 and AML2 primary samples, respectively, compared to 6.01 nM for MOLM-13 cells and 1.87 nM for MV4-11 cells, indicating that this assay may require higher doses than would be necessary clinically. We hypothesize that this is due to slower proliferation rates in an ex vivo context and the requirement of a support system (here, HS5 cells) to maintain an adequate viability to complete the experiment. Thus, we conclude that while the synergy was demonstrably lower and required higher dose ranges, the presence of any synergy in primary patient samples in an ex vivo context supports further study of the combination of drugs.

### 2.4. Combination Therapy with Midostaurin or Gilteritinib and Selinexor Prolongs Survival in a Human Xenograft Model of AML

**Figure 4:**
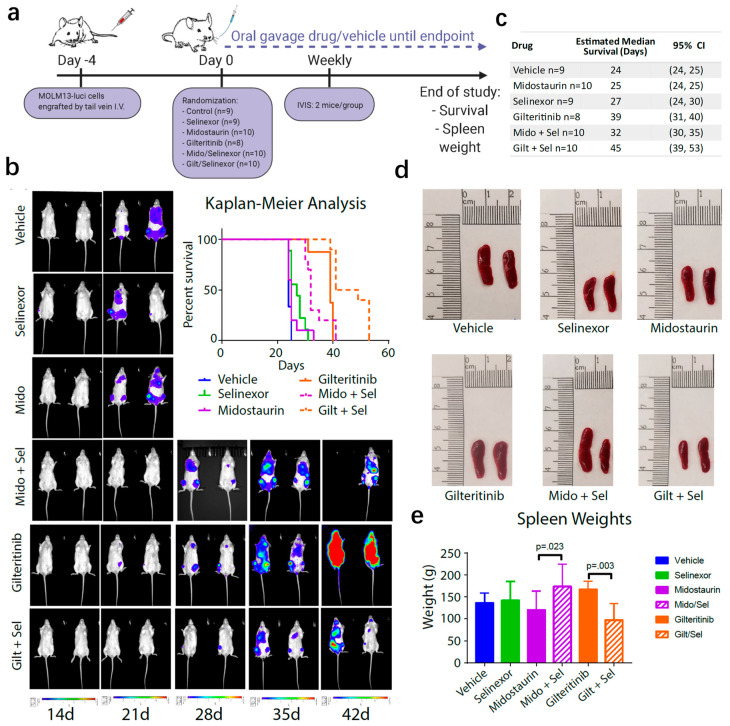
In vivo pharmacologic validation of XPO1 as a cotarget with FLT3. (**a**) Design of murine experiment where NSG mice were engrafted with MOLM-13 cells expressing luciferase and treated with vehicle, 50 mg/kg midostaurin daily, 30 mg/kg gilteritinib daily, 15 mg/kg selinexor twice weekly, or combinations of midostaurin or gilteritinib plus selinexor. (**b**) IVIS imaging of two mice per group and Kaplan-Meier analysis of survival of the entire cohort. (**c**) Estimated mean survival for each group with the 95% confidence interval (CI). (**d**) Representative images of spleens from each group ex vivo. (**e**) Differences in spleen weights between groups were estimated using analysis of variance (ANOVA) methods. *p*-values were adjusted for multiple comparisons within each drug combination using Holm’s procedure.

The promising in vitro results led us to evaluate the in vivo efficacy in an aggressive MOLM-13 xenograft mouse model. Human MOLM-13 cells that express luciferase to allow for visualization of disease burden and dissemination were engrafted into NOD-*Prkdc^em26Cd52^Il2rg^em26Cd22^*/NjuCrl (NCG) mice. Mice were treated with 30 mg/kg gilteritinib daily, 50 mg/kg midostaurin daily, 15 mg/kg selinexor twice weekly, combinations of gilteritinib or midostaurin with selinexor, or vehicle control (Figure 4a). IVIS imaging and Kaplan–Meier survival curves indicate that combination therapy extended survival beyond its constituent monotherapies or vehicle (Figure 4b). Estimated median survival of midostaurin plus selinexor was 32 days compared to 24 days for vehicle (*p* < 0.001), 25 days for midostaurin (*p* = 0.001), and 27 days for selinexor (*p* < 0.001; Figure 4c). Estimated median survival of gilteritinib plus selinexor was 45 days compared to 24 days for vehicle (*p* < 0.001), 27 days for selinexor (*p* < 0.001), and 39 days for gilteritinib (*p* < 0.001). Spleen size and weight did not appear to be dramatically different for the majority of conditions (Figure 4d,e). However, mice given gilteritinib and selinexor had lower spleen weights compared to gilteritinib alone (*p* = 0.003). Together, this data suggests that this combination could prove beneficial for AML patients harboring *FLT3*-ITD and merits further clinical development.

## 3. Discussion

*FLT3*-ITD mutations represent the most common type of AML that confers poor prognosis in AML, even when it occurs with favorable mutations such as NPM1. Strategies targeting FLT3 activation either in combination with chemotherapy using the first-generation inhibitor midostaurin or monotherapy with the more potent and selective inhibitor gilteritinib have shown favorable clinical outcome and improved survival but still a very low rate of cure. Given this observation, we hypothesized that FLT3 inhibitor therapy with either midostaurin or gilteritinib given as monotherapy or in combination with chemotherapy will be insufficient for producing a lasting remission. We therefore sought to determine if these drugs could be combined with an additional targeted drug approved for marketing by the FDA to overcome limitations such as short duration of response and relapse. To accomplish this, we utilized the novel technique of CRISPR knockout screening to provide a genome-wide, unbiased approach to identifying genes whose knockout is predicted to enhance the antileukemic effects of FLT3 inhibitors. Doing this, we were able to identify the gene nuclear exporter XPO1 as well as many genes involved in the nuclear pore, including interacting partners of XPO1. Given the prediction from our screen that XPO1 and FLT3 inhibitors would synergistically exhibit robust antileukemic activity when in combination—which was strengthened by pathway analysis—we pursued genetic target validation by knocking down expression of XPO1 protein levels. This demonstrated that while XPO1 knockdown alone, midostaurin treatment alone, or gilteritinib treatment alone were all capable of decreasing viable cells, the combination of XPO1 knockdown with either midostaurin or gilteritinib resulted in greater reduction of viable cells compared to each condition alone or vehicle control. Similarly, our data showed that inhibition of XPO1 via the slowly reversible inhibitor selinexor phenocopied the effect of CRISPR-mediated XPO1 KD. Specifically, in vitro cell line, primary cell experiments and xenograft studies combining these two classes of drugs demonstrated synergistic benefit. Collectively, this provides a therapeutic pathway for novel combination therapies using XPO1 inhibitors together with FLT3 inhibitors.

XPO1 (Exportin-1) has been recognized as a potential therapeutic target in multiple tumor types, including AML, and the magnitude of its overexpression at an mRNA and protein level has been directly correlated with poor overall survival. Development of XPO1 inhibitors for clinical use has been somewhat challenging due to the modest therapeutic window that exists with current therapeutic agents. Selinexor was the first slowly reversible XPO1 inhibitor to enter clinical development and is currently marketed for third line treatment of multiple myeloma in combination with dexamethasone. Preclinical studies from several groups have demonstrated that selinexor (KPT-330) or its second-generation molecule eltanexor (KPT-8602) have in vitro and in vivo activity in AML preclinical models and inhibit leukemia initiating stem cells. Studies have demonstrated synergy with other agents including topoisomerase 2 inhibitors, BCL-2 inhibitors, hypomethylating agents, and broad kinase inhibitors that include FLT3 (sorafinib) [11]. Additionally, one study of diagnosis-to-relapse *FLT3*-ITD patients demonstrated that XPO1 expression increased at time of relapse and inhibition of XPO1 with selinexor increased sensitivity to induction chemotherapy used for AML. In a phase I clinical trial of selinexor monotherapy for AML patients examining multiple doses, 9% of patients responded to treatment at various doses [8]. Interestingly, the observed decrease in FLT3 protein expression following selinexor treatment appeared to be a post-translational effect, with no change in mRNA levels of the same samples. Inferentially, these data likely suggest a favorable interaction of FLT3 and XPO1 inhibition in *FLT3*-ITD-positive AML. Our unbiased CRISPR-Cas9 screening data and validation with two distinct chemical entities that inhibit FLT3 in AML cell lines and primary cells provides further evidence for this. Indeed, not only KD of XPO1, but also other members of this nuclear export complex were identified. Although more laborious, the application of CRISPR-Cas9 screening, which examines targets for drug synergy in a potentially unbiased and comprehensive manner, adds to the molecular pharmacologic and in vivo tools that can be utilized by drug development teams to assure the best combination strategies and drugs are utilized in clinical trials. Collectively, these data suggest a potential role for combination strategies of FLT3 and XPO1 inhibitors in clinical trials.

Clinical development of the combination of a FLT3 and XPO1 inhibitor should consider both overlapping toxicities and experience in development of each therapeutic as a monotherapy. XPO1 inhibitors such as selinexor may cause distinct gastrointestinal and constitutional symptoms that in AML patients were problematic above the 40 mg/m^2^ dosing used in the phase 1 AML study. Even for lower doses of selinexor, these symptoms can occur. Gilteritinib is a more selective and potent inhibitor of FLT3 that is approved as monotherapy for relapsed AML and generally lacks these gastrointestinal and constitutional symptoms. In contrast, midostaurin is a less specific and potent FLT3 inhibitor approved only in combination therapy with chemotherapy and has significant gastrointestinal symptoms associated with it. A similar earlier study observed that the less specific and potent FLT3 inhibitor sorafenib synergizes with selinexor [11], but likely will have the same challenges for overlapping toxicities of the two agents. Furthermore, with sorafinib, there are abundant other alternative targets inhibited. While we did not test the toxic effects on normal hematopoietic stem cells, we expect it would be minimal because these cells do not have a FLT3 mutation and are therefore less affected by FLT3 inhibitors. Additionally, it has been previously shown that selinexor and XPO1 inhibition does not affect normal hematopoietic stem cells [10,27]. Moving forward with combination strategies, both the preclinical data demonstrating superiority of selinexor together with gilteritinib as compared to midostaurin support this combination. Additionally, given the absence of significant gastrointestinal complications with gilteritinib, we would expect better tolerability of this combination therapy. Efforts to initiate such a trial of gilteritinib and selinexor are underway at this time.

## 4. Materials and Methods

### 4.1. Cell Culture

MOLM-13 and MV4-11 cell lines were purchased from Deutsche Sammlung von Mikroorganismen und Zellkulturen (Braunschweig, Germany) in February 2015. Our targeted sequencing panel and capillary gel electrophoresis indicated that MOLM-13 cells have variants in *NF1*, *FLT3,* and *CBL*; MV4-11 cells were found to have variants in *TP53* and *FLT3*. HEK293FT cells were purchased from Life Technologies (R70007) (Carlsbad, CA, USA). Cells were propagated in filtered RPMI 1640 (Gibco; Waltham, MA, USA) for MOLM-13, MV4-11, and HS5 cell lines or DMEM (Gibco) for HEK293FT cells. All media were supplemented with 10% Fetal Bovine Serum (FBS, VWR; Radnor, PA, USA) and 1% penicillin/streptomycin/glutamine. HS5-GFP cells were kindly gifted from Dr. William Dalton (H. Lee Moffitt Cancer Center) and MOLM-13-luciferase cells from Dr. Ramiro Garzon (Ohio State University). Cell lines were validated by microsatellite genotyping (short tandem repeat analysis by the Ohio State University Genomic Services Core), routinely tested negative for mycoplasma contamination (Universal Mycoplasma Detection Kit, ATCC 30-1012K), and were discarded after passage twenty.

### 4.2. Genome-Wide Loss-of-Function Screening

The human Brunello CRISPR knockout pooled library was a gift from David Root and John Doench (Addgene #73178; Watertown, MA, USA) [12]. The library was amplified and lentiviral particles were produced as described in provided protocols on Addgene. Briefly, Brunello library plasmids were amplified in NEB 5-alpha electrocompetent cells and plated on LB agar/ampicillin bioassay plates. Samples were prepared with endotoxin-free MaxiPrep (Qiagen; Hilden, Germany) as per the manufacturer’s protocol. Plasmid DNA was packaged into viral particles in HEK293FT cells. In prescreen experiments, plasmid DNA was PCR-amplified, sequenced on an Illumina MiSeq, and analyzed with MAGeCK [13] to ensure minimal loss of diversification. Viral particle infection efficiency and optimal volume of viral particles for addition was determined with a small-scale spin infection and treatment with puromycin. Optimal puromycin and polybrene concentrations for MOLM-13 cells, were determined empirically to be 2 µg/mL and 10 µg/mL, respectively.

For screening, 2.5 × 10^6^ cells/well were plated in each well of 12-well plates with total cells equal to 400 cells/sgRNA × 76,441 sgRNAs × 100/infection efficiency (%) to determine the total cells per condition. Media containing lentiviral particles was added at the predetermined ratio with polybrene and spinoculated at 450× *g* for 90 min. After six hours, cells were centrifuged 300× *g* for 10 min and resuspended in complete media. Puromycin selection was started after three days and continued for seven days of selection to remove essential and nontransduced cells. Cultures were then exposed to 30 nM midostaurin (Selleck), or dimethyl sulfoxide (DMSO, 0.00012%) as a vehicle control for three days. Cells were harvested, DNA extracted via Qiagen Blood & Cell Culture DNA Midi Kit per manufacturer’s instructions, and the sgRNA cassette retrieved via PCR as described by Broad sequencing protocol on Addgene. The P5 mix and P7 primers design from this protocol were used with the following New England Biolabs NEBNext Indexes: 17, 24, 26, 28, 29, 31, 33, 34, 35, 36, and 42. Sample sequencing on an Illumina HiSeq4000 was performed at the Institute for Genomic Medicine at Nationwide Children’s Hospital, Columbus, Ohio.

Analysis of resultant FASTQ utilized Snakemake [28] to organize a pipeline for analysis, skewer [29] to trim, and MAGeCK [13,30,31] for count and robust rank algorithm analysis. Postpuromycin selection levels of sgRNA were compared to plasmid levels for essential gene analysis of MOLM-13 cells. For drugging studies, post-treatment levels of sgRNA were normalized to postpuromycin selection (pretreatment) levels. Vehicle arms allowed for assessment of late essential genes and mitigated effects of amplified regions of DNA on false positives [32,33].

### 4.3. CRISPR Ribonucleoprotein Knockout

Following manufacturer protocols, ribonucleoprotein (RNP) complexes were formed using Alt-R CRIPSR-Cas9 cRNA, tracrRNA, and Cas9 (Integrated DNA Technologies; Coralville, IA, USA). RNP and Alt-R Cas9 electroporation enhancer were added to MOLM-13 cells and electroporated in Solution SF (Lonza V4XC-2012) on a Lonza 4D-Nucleofector System with pulse code EH100. The following Alt-R CRISPR–Cas9 sgRNAs targeting XPO1 were used as a pool: GGTTGAAACCGGTTCAGACT, GAGAGGGGACGAATCAAGGT, and TCCCAAGCTCTCCACCGAGG.

### 4.4. Immunoblot Analysis

Extracted proteins from whole-cell lysates were resolved by SDS–PAGE, then transferred onto nitrocellulose membrane as previously described [34]. Blots were probed with anti-GAPDH (Millipore MAB374; Burlington, MA, USA) and anti-XPO1 (Novus NB-100) antibodies. Western blots were developed on film as well as quantified on a BioRad ChemiDoc imager and using ImageJ software (Figure S5) [35].

### 4.5. Proliferation Assay and Flow Cytometric Studies

Cell proliferation and death were determined via MTS and Annexin V-FITC flow-based assays as previously described [34].

### 4.6. Primary AML Sample Proliferation Assays

*FLT3*-ITD cryopreserved primary cells collected during apheresis of AML patients with different co-occurring mutations (Figure S4) were obtained from the Ohio State University Comprehensive Cancer Center Leukemia Tissue Bank. For proliferation assays, 1 × 10^5^ cells in 96-well plates were plated with HS5-GFP stromal cells and incubated in a range of drug doses. CellTiter 96 (Promega; Madison, WI, USA) was added after 96 h and incubated an additional five hours. Leukemic cells were then transferred to a new plate and formazan dye was quantified by absorbance (490 nm). The dose range of midostaurin had to be limited because doses greater than 100 nM caused stromal detachment. A next-generation sequencing targeted capture panel was used to determine mutational status of samples, which was visualized with Oncoprint [36,37].

### 4.7. Animal Studies

Animal experiments were carried out under protocols approved by the Ohio State University: protocol number 2015A00000043-R1 and OLAW assurance A3261-01. Institutional Animal Care and Use Committee. A total of 10^4^ MOLM-13 luciferase-tagged cells were injected into the tail vein of NOD-*Prkdc^em26Cd52^Il2rg^em26Cd22^*/NjuCrl (NCG) mice from the Charles River Laboratory. Three days postengraftment, mice begin treatment of 50 mg/kg midostaurin (MedChemExpress HY-10230; Monmouth Junction, NJ, USA) daily gavage in 6% drug *w/w* gelucire(R)44/14 (Gattefosse; Montville, NJ, USA), which was aliquoted and mixed with drug weekly; 30 mg/kg gilteritinib (MedChemExpress HY-12432) daily gavage in 6% drug *w/w* gelucire(R)44/14 (Gattefosse, France), which was aliquoted and mixed with drug weekly; 15 mg/kg selinexor (KPT-330, Karyopham Therapeutics; Newton, MA, USA) gavage in 0.6% *w/v* pluronic F-68 (Life Technologies 24040032) and 0.6% *w/v* plasdone K-29/32 (Sigma-Aldrich 234257; St. Louis, MO, USA) on two consecutive days every week; or combinations thereof. Aliquots of gelucire were stored at 4 °C and warmed each day to 44 °C in a heat block, then diluted with sterile water to a concentration of 12 mg/mL.

Appropriate dose was determined daily based on weight with a maximum volume of 200 µL for oral gavage. Animal technicians who were blinded to treatment groups routinely monitored mice for Early Removal Criteria (20% weight loss, lethargy, pallor, labored breathing, hunching, poor body condition). At 10% weight loss, mice were given a two-day drug holiday.

### 4.8. Statistics

For CRISPR screening, statistics were calculated using MAGeCK software [31]. In combination proliferation assays, synergistic interactions were determined using Combenefit software [38] employing the Highest Single Agent model [39], which determines at what doses the combination effect is more than the single agent with the greatest effect. Mathematical synergy was established by showing that the decrease in proliferation (relative to DMSO) was mathematically greater than the sum of the individual effects. That is: [ln(combination) − ln(DMSO)] < [ln(FLT3 inhibitor) − ln(DMSO)] + [ln(selinexor) − ln(DMSO)]. A linear mixed effects model was applied to the log-transformed data to allow for correlations among observations from the same biological replicate/patient.

The mathematical synergy and all other statistical analyses aside from those described above were performed using SAS/STAT software, version 9.4 of the SAS System for Windows (SAS Institute, Inc., Cary, NC, USA). For XPO1 KD experiments, dependencies among observations from the same biological replicate and the interaction between parent/KD and drug were accounted for using mixed effects models. For CFU assays, differences between conditions were assessed after fitting negative binomial models to the count data. The primary endpoint of mouse survival experiments was overall survival (OS). Kaplan–Meier methods were used to estimate median survival time in each group. The log-rank test was used to assess differences in OS between groups. For spleen weight, differences between groups were estimated using analysis of variance (ANOVA) methods, and *p*-values were adjusted for multiple comparisons within each drug combination using Holm’s procedure.

### 4.9. Data Sharing Statement

All sequence data are available in the Gene Expression Omnibus via accession number GSE143314.

## 5. Conclusions

Using a large, unbiased CRISPR-Cas9 knockout screen we identified many genes whose knockout is predicted to enhance the antileukemic effect of FLT3 inhibitors midostaurin and gilteritinib. Among these genes we selected XPO1 for further validation because of the potential for rapid clinical translation of combination therapies of gilteritinib with FDA-approved XPO1 inhibitor selinexor to augment antileukemic activity.

Knockdown of protein levels of XPO1 with midostaurin or gilteritinib treatment reproduced the synergy demonstrated during the genome-wide screening. Replacement of genetic perturbation of XPO1 with selinexor also reproduced the expected synergy. Furthermore, the combinations of midostaurin and selinexor or gilteritinib and selinexor were effective across *FLT3*-ITD AML cell lines, primary patient samples, and in an aggressive xenograft mouse model. This work shows the power of using large CRISPR screens to identify combination therapies relevant to a particular targeted therapy for translation to the clinic, demonstrates how pathway analysis can be used to strengthen predictive power of the screens, and uses multiple orthogonal approaches to validate the predicted synergy. Thus, this work serves as a template for development of rational combination strategies using CRISPR screening and—in application to the particular case of FLT3 inhibitors—supports the further clinical development of cotherapy with FLT3 inhibitors, especially gilteritinib and selinexor.

## Figures and Tables

**Table 1:** XPO1 cargo proteins. XPO1 binding proteins were mined from literature and using NESdb. All cargo proteins that demonstrated statistical significance (*p* < 0.01 and FDR < 0.25) are listed with their log-fold-change (LFC), *p*-value, and false discovery rate (FDR).

Gene Symbol	Gene Name	LFC	*p*-Value	FDR
KPNB1	Karyopherin subunit beta 1	−2.0259	2.59 × 10^−7^	0.000171
BIRC5	Baculoviral IAP Repeat Containing 5	−1.4075	4.95 × 10^−5^	0.005873
MEN1	Menin 1	−1.1829	0.000111	0.010163
COPS5	COP9 signalosome subunit 5	−1.169	0.000201	0.013421
NUP88	Nucleoporin 88	−1.1078	3.91 × 10^−5^	0.005085
WEE1	WEE 1 G2 checkpoint kinase	−1.10108	0.000128	0.010989
PCNA	Proliferting cell nuclear antigen	−0.93675	0.000216	0.013421
RanGAP1	RanGTPase-activating protein	−0.92319	3024 × 10^−5^	0.004428
HDAC3	Histone deacetylase 3	−0.84395	3.29 × 10^−5^	0.004428
HSPA8	Heat shock protein family A (Hsp70) member 8	−0.79534	8.42 × 10^−5^	0.008424
CDC7	Cell division cycle 7	−0.78328	0.000117	0.010461
NUP214	Nucleoporin 214	−0.74563	0.00453	0.091762
AK6	Adenylate kinase 6	−0.63239	0.000849	0.030247
FANCA	FA complementation group A	−0.60663	0.000502	0.02192
PCYT1A	Phosphate cytidylyltransferase, choline, alpha	−0.59981	0.000639	0.025306
TOP2A	DNA topoisomerase II alpha	−0.5615	0.008611	0.116464
RAN	RAN, member RAS oncogene family	−0.5343	1.81 × 10^−6^	0.000737
NPM1	Nucleophosmin 1	−0.47427	0.000517	0.02192
GNL3L	G protein nucleolar 3 like	−0.42729	0.006196	0.110075
BECN1	Beclin 1	−0.42416	0.002697	0.067135
STRADA	STE20 related adaptor alpha	−0.38892	0.003048	0.074302
MCM3	Minichromosome maintenance complex component 3	−0.38083	0.008611	0.116464
IPMK	Inositol polyphosphate multikinase	−0.34517	0.001961	0.055281
DDX6	DEAD-box helicase 6	−0.30465	0.004211	0.090461
TGM2	Transglutaminase 2	−0.1821	0.009599	0.121196
DIAPH3	Diaphanous related formin 3	−0.05134	0.008152	0.116464

## Supplementary Materials

The following are available online at https://www.mdpi.com/2072-6694/12/6/1574/s1, Figure S1: Quality metrics of midostaurin CRISPR screen, Figure S2: Mapping of CRISPR screening results onto AML signaling pathways, Figure S3: Additional analyses of synergy from combination proliferation assays, Figure S4: Primary samples used in MTS assays, Figure S5: Immunoblot analysis, Table S1: Top Hits from Midostaurin Screen.

## References

[1] Döhner H., Estey E., Grimwade D., Amadori S., Appelbaum F.R., Büchner T., Dombret H., Ebert B.L., Fenaux P., Larson R.A. (2017). Diagnosis and management of AML in adults: 2017 ELN recommendations from an international expert panel. Blood.

[2] Schlenk R.F., Kayser S., Bullinger L., Kobbe G., Casper J., Ringhoffer M., Held G., Brossart P., Lübbert M., Salih H.R. (2014). Differential impact of allelic ratio and insertion site in *FLT3*-ITD-positive AML with respect to allogeneic transplantation. Blood.

[3] Garg M., Nagata Y., Kanojia D., Mayakonda A., Yoshida K., Haridas Keloth S., Zang Z.J., Okuno Y., Shiraishi Y., Chiba K. (2015). Profiling of somatic mutations in acute myeloid leukemia with *FLT3*-ITD at diagnosis and relapse. Blood.

[4] Stone R.M., Mandrekar S.J., Sanford B.L., Laumann K., Geyer S., Bloomfield C.D., Thiede C., Prior T.W., Döhner K., Marcucci G. (2017). Midostaurin plus Chemotherapy for Acute Myeloid Leukemia with a FLT3 Mutation. N. Engl. J. Med..

[5] Zhao J., Song Y., Liu D. (2019). Gilteritinib: A novel FLT3 inhibitor for acute myeloid leukemia. Biomark. Res..

[6] Bazarbachi A., Bug G., Baron F., Brissot E., Ciceri F., Dalle I.A., Döhner H., Esteve J., Floisand Y., Giebel S. (2020). Clinical practice recommendation on hematopoietic stem cell transplantation for acute myeloid leukemia patients with FLT3-internal tandem duplication: A position statement from the Acute Leukemia Working Party of the European Society for Blood and Marrow Transplantation. Haematologica.

[7] Ranganathan P., Yu X., Na C., Santhanam R., Shacham S., Kauffman M., Walker A., Klisovic R., Blum W., Caligiuri M. (2012). Preclinical activity of a novel CRM1 inhibitor in acute myeloid leukemia. Blood.

[8] Garzon R., Savona M., Baz R., Andreeff M., Gabrail N., Gutierrez M., Savoie L., Mau-Sorensen P.M., Wagner-Johnston N., Yee K. (2017). A phase 1 clinical trial of single-agent selinexor in acute myeloid leukemia. Blood.

[9] Kojima K., Kornblau S.M., Ruvolo V., Dilip A., Duvvuri S., Davis R.E., Zhang M., Wang Z., Coombes K.R., Zhang N. (2013). Prognostic impact and targeting of CRM1 in acute myeloid leukemia. Blood.

[10] Etchin J., Montero J., Berezovskaya A., Le B., Kentsis A., Christie A., Conway A., Chen W., Reed C., Mansour M. (2016). Activity of a selective inhibitor of nuclear export, selinexor (KPT-330), against AML-initiating cells engrafted into immunosuppressed NSG mice. Leukemia.

[11] Zhang W., Ly C., Ishizawa J., Mu H., Ruvolo V., Shacham S., Daver N., Andreeff M. (2018). Combinatorial targeting of XPO1 and FLT3 exerts synergistic anti-leukemia effects through induction of differentiation and apoptosis in FLT3-mutated acute myeloid leukemias: From concept to clinical trial. Haematologica.

[12] Doench J.G., Fusi N., Sullender M., Hegde M., Vaimberg E.W., Donovan K.F., Smith I., Tothova Z., Wilen C., Orchard R. (2016). Optimized sgRNA design to maximize activity and minimize off-target effects of CRISPR-Cas9. Nat. Biotechnol..

[13] Wang B., Wang M., Zhang W., Xiao T., Chen C.-H., Wu A., Wu F., Traugh N., Wang X., Li Z. (2019). Integrative analysis of pooled CRISPR genetic screens using MAGeCKFlute. Nat. Protoc..

[14] Connor M.K., Kotchetkov R., Cariou S., Resch A., Lupetti R., Beniston R.G., Melchior F., Hengst L., Slingerland J.M. (2003). CRM1/Ran-Mediated Nuclear Export of p27Kip1 Involves a Nuclear Export Signal and Links p27 Export and Proteolysis. Mol. Biol. Cell.

[15] Dong X., Biswas A., Süel K.E., Jackson L.K., Martinez R., Gu H., Chook Y.M. (2009). Structural basis for leucine-rich nuclear export signal recognition by CRM1. Nature.

[16] Lindsay M.E., Holaska J.M., Welch K., Paschal B.M., Macara I.G. (2001). Ran-binding protein 3 is a cofactor for Crm1-mediated nuclear protein export. J. Cell Biol..

[17] Daelemans D., Costes S.V., Lockett S., Pavlakis G.N. (2005). Kinetic and molecular analysis of nuclear export factor CRM1 association with its cargo in vivo. Mol. Cell. Biol..

[18] Cheng Y., Holloway M.P., Nguyen K., McCauley D., Landesman Y., Kauffman M.G., Shacham S., Altura R.A. (2014). XPO1 (CRM1) inhibition represses STAT3 activation to drive a survivin-dependent oncogenic switch in triple-negative breast cancer. Mol. Cancer Ther..

[19] Xu D., Grishin N.V., Chook Y.M. (2012). NESdb: A database of NES-containing CRM1 cargoes. Mol. Biol. Cell.

[20] Ma J., Zhao S., Qiao X., Knight T., Edwards H., Polin L., Kushner J., Dzinic S.H., White K., Wang G. (2019). Inhibition of Bcl-2 Synergistically Enhances the Antileukemic Activity of Midostaurin and Gilteritinib in Preclinical Models of FLT3-mutated Acute Myeloid Leukemia. Clin. Cancer Res..

[21] Perl A.E., Daver N.G., Pratz K.W., Maly J., Hong W.-J., Bahceci E., Tong B., Tian T., Dilley K. (2019). Venetoclax in Combination with Gilteritinib in Patients with Relapsed/Refractory Acute Myeloid Leukemia: A Phase 1b Study. Blood.

[22] Kanehisa M., Goto S. (2000). KEGG: Kyoto Encyclopedia of Genes and Genomes. Nucleic Acids Res..

[23] McMahon C.M., Ferng T., Canaani J., Wang E.S., Morrissette J.J., Eastburn D.J., Pellegrino M., Durruthy-Durruthy R., Watt C.D., Asthana S. (2019). Clonal selection with Ras pathway activation mediates secondary clinical resistance to selective FLT3 inhibition in acute myeloid leukemia. Cancer Discov..

[24] Perl A.E., Martinelli G., Cortes J.E., Neubauer A., Berman E., Paolini S., Montesinos P., Baer M.R., Larson R.A., Ustun C. (2019). Gilteritinib or Chemotherapy for Relapsed or Refractory FLT3-Mutated AML. N. Engl. J. Med..

[25] Fischer T., Stone R.M., Deangelo D.J., Galinsky I., Estey E., Lanza C., Fox E., Ehninger G., Feldman E.J., Schiller G.J. (2010). Phase IIB trial of oral Midostaurin (PKC412), the FMS-like tyrosine kinase 3 receptor (FLT3) and multi-targeted kinase inhibitor, in patients with acute myeloid leukemia and high-risk myelodysplastic syndrome with either wild-type or mutated FLT3. J. Clin. Oncol..

[26] Perl A.E., Altman J.K., Cortes J., Smith C., Litzow M., Baer M.R., Claxton D., Erba H.P., Gill S., Goldberg S. (2017). Selective Inhibition of FLT3 by Gilteritinib in Relapsed/Refractory Acute Myeloid Leukemia: A Multicenter, First-in-human, Open-label, Phase 1/2 Study. Lancet Oncol..

[27] Etchin J., Berezovskaya A., Conway A.S., Galinsky I.A., Stone R.M., Baloglu E., Senapedis W., Landesman Y., Kauffman M., Shacham S. (2017). KPT-8602, a second-generation inhibitor of XPO1-mediated nuclear export, is well tolerated and highly active against AML blasts and leukemia-initiating cells. Leukemia.

[28] Köster J., Rahmann S. (2012). Snakemake—A scalable bioinformatics workflow engine. Bioinformatics.

[29] Jiang H., Lei R., Ding S.-W., Zhu S. (2014). Skewer: A fast and accurate adapter trimmer for next-generation sequencing paired-end reads. BMC Bioinform..

[30] Li W., Xu H., Xiao T., Cong L., Love M.I., Zhang F., Irizarry R.A., Liu J.S., Brown M., Liu X.S. (2014). MAGeCK enables robust identification of essential genes from genome-scale CRISPR/Cas9 knockout screens. Genome Biol..

[31] Li W., Köster J., Xu H., Chen C.-H., Xiao T., Liu J.S., Brown M., Liu X.S. (2015). Quality control, modeling, and visualization of CRISPR screens with MAGeCK-VISPR. Genome Biol..

[32] Aguirre A.J., Meyers R.M., Weir B.A., Vazquez F., Zhang C.-Z., Ben-David U., Cook A., Ha G., Harrington W.F., Doshi M.B. (2016). Genomic Copy Number Dictates a Gene-Independent Cell Response to CRISPR/Cas9 Targeting. Cancer Discov..

[33] Meyers R.M., Bryan J.G., McFarland J.M., Weir B.A., Sizemore A.E., Xu H., Dharia N.V., Montgomery P.G., Cowley G.S., Pantel S. (2017). Computational correction of copy number effect improves specificity of CRISPR-Cas9 essentiality screens in cancer cells. Nat. Genet..

[34] Ozer H.G., El-Gamal D., Powell B., Hing Z.A., Blachly J.S., Harrington B., Mitchell S., Grieselhuber N.R., Williams K., Lai T.-H. (2018). BRD4 Profiling Identifies Critical Chronic Lymphocytic Leukemia Oncogenic Circuits and Reveals Sensitivity to PLX51107, a Novel Structurally Distinct BET Inhibitor. Cancer Discov..

[35] Schneider C.A., Rasband W.S., Eliceiri K.W. (2012). NIH Image to ImageJ: 25 years of image analysis. Nat. Methods.

[36] Cerami E., Gao J., Dogrusoz U., Gross B.E., Sumer S.O., Aksoy B.A., Jacobsen A., Byrne C.J., Heuer M.L., Larsson E. (2012). The cBio cancer genomics portal: An open platform for exploring multidimensional cancer genomics data. Cancer Discov..

[37] Gao J., Aksoy B.A., Dogrusoz U., Dresdner G., Gross B., Sumer S.O., Sun Y., Jacobsen A., Sinha R., Larsson E. (2013). Integrative analysis of complex cancer genomics and clinical profiles using the cBioPortal. Sci. Signal..

[38] Di Veroli G.Y., Fornari C., Wang D., Mollard S., Bramhall J.L., Richards F.M., Jodrell D.I. (2016). Combenefit: An interactive platform for the analysis and visualization of drug combinations. Bioinformatics.

[39] Berenbaum M.C. (1989). What is synergy?. Pharmacol. Rev..

