# Supplementary material for: Cotargeting of XPO1 Enhances the Antileukemic Activity of Midostaurin and Gilteritinib in Acute Myeloid Leukemia
Source: Cancers (Basel). 2020 Jun 14;12(6):1574. doi: 10.3390/cancers12061574 (PMC7352446; doi:10.3390/cancers12061574)
Supplement: Supplementary file 1 [file cancers-12-01574-s001.pdf]

Supplemental Figures

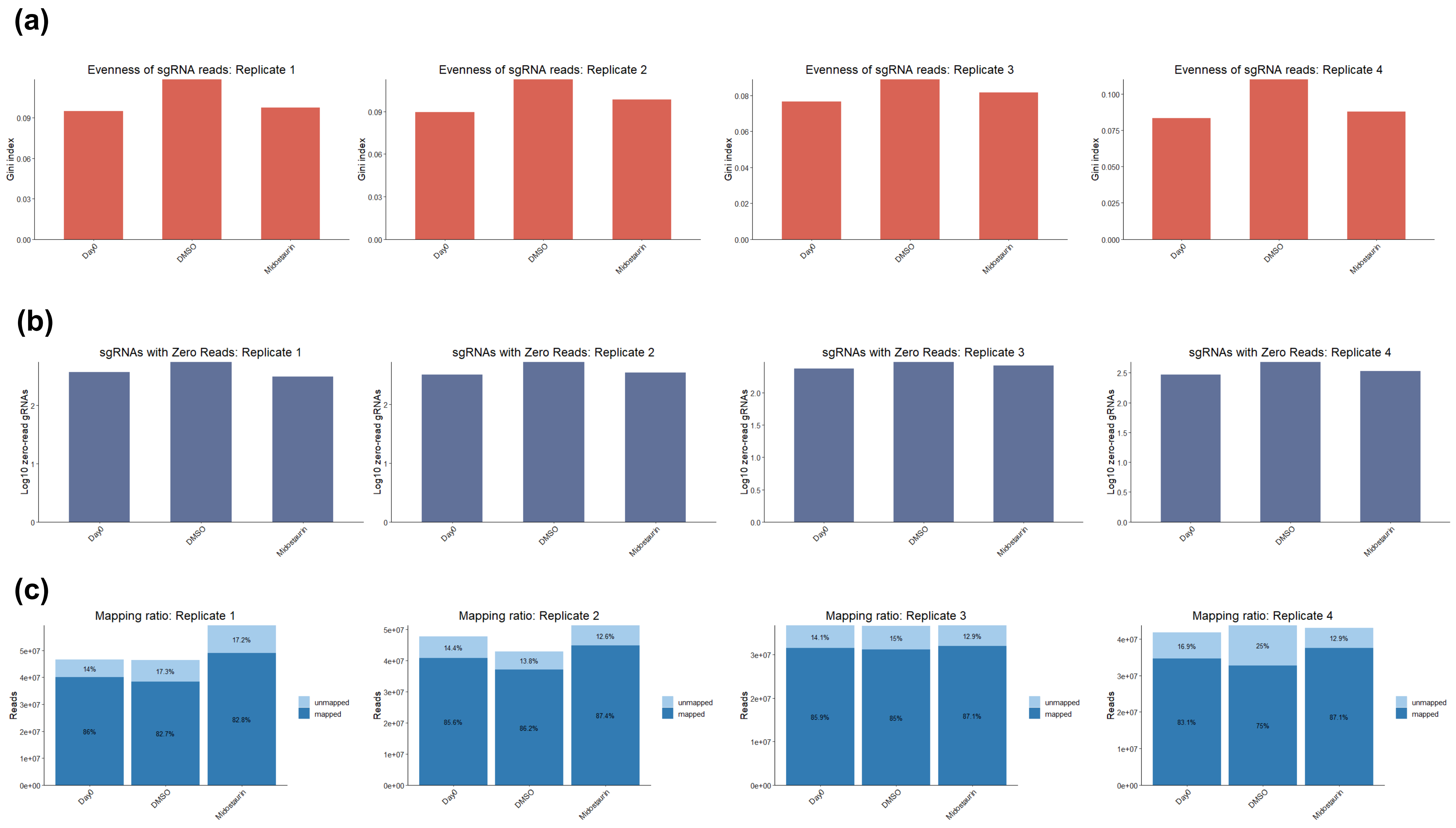

Figure S1: Quality metrics of midostaurin CRISPR screen. (a) Evenness of sgRNA reads, (b) Number of sgRNAs with zero reads, and (c) Mapping ratio of sgRNAs to those present in library in each condition and replicate.

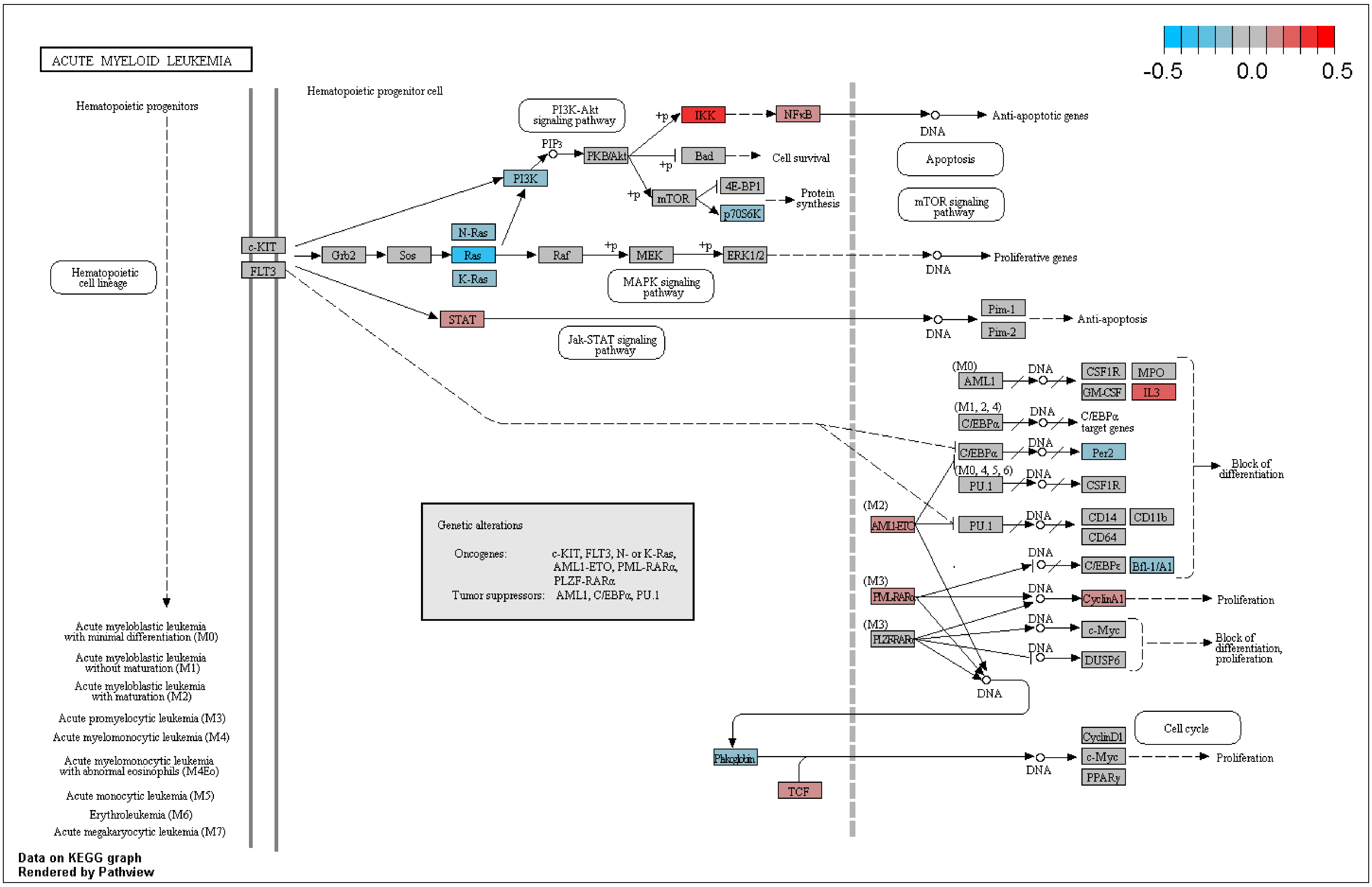

Figure S2: Mapping of CRISPR screening results onto AML signaling pathways. Visualization of positively- (red) and negatively-selected (blue) gene KOs on Acute Myeloid Leukemia FLT3 signaling map. Strength of selection is represented by color saturation.

Midostaurin Screen Hit	LFC	P-value	FDR
CDK6	-2.6692	2.59E-07	0.000171
RPS8	-2.3407	2.59E-07	0.000171
BCL2	-2.2782	2.59E-07	0.000171
SPDYE2B	-2.0816	7.77E-07	0.000495
KPNB1	-2.0259	2.59E-07	0.000171
SNRPA1	-1.9994	1.81E-06	0.000737
RPL36	-1.9808	2.33E-06	0.000768
RPS20	-1.9661	2.59E-07	0.000171
TRMT112	-1.9645	2.59E-07	0.000171
RFC3	-1.853	2.59E-07	0.000171
UBA52	-1.8434	2.85E-06	0.000778
COPE	-1.8203	3.37E-06	0.000825
POLR2I	-1.6784	1.81E-06	0.000737
SNRPD3	-1.6589	1.29E-06	0.000737
GART	-1.6383	2.59E-07	0.000171
RPS10	-1.5933	3.37E-06	0.000825
RPL15	-1.5769	2.33E-06	0.000768
PAICS	-1.5734	4.40E-06	0.001039
DNAJC9	-1.5542	7.51E-06	0.001495
SRSF2	-1.548	5.15E-05	0.006081
HGC6.3	-1.5441	2.56E-05	0.004017
POLE	-1.5384	1.81E-06	0.000737
DUT	-1.5375	2.85E-06	0.000778
INTS1	-1.4944	4.92E-06	0.001069
USP17L25	-1.4745	8.11E-05	0.008331
LOC102724862	-1.4735	1.74E-05	0.003043
PTPN11	-1.4296	2.59E-07	0.000171
NBPF20	-1.4093	7.51E-06	0.001495
BIRC5	-1.4075	4.95E-05	0.005873
RPL3	-1.3954	0.00017844	0.013421
TBCC	-1.3953	1.81E-06	0.000737

Table S1: Top Hits from Midostaurin Screen.

(a)

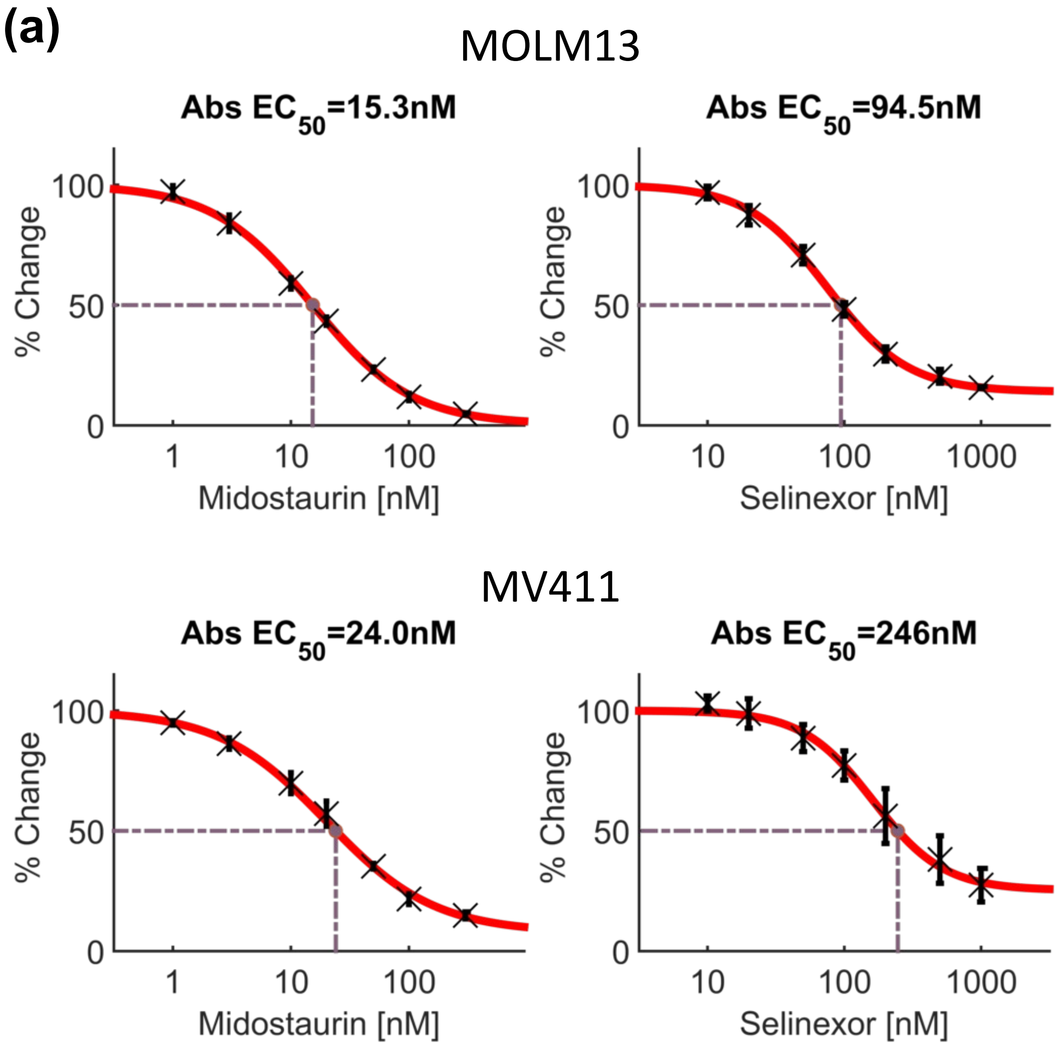

(b)

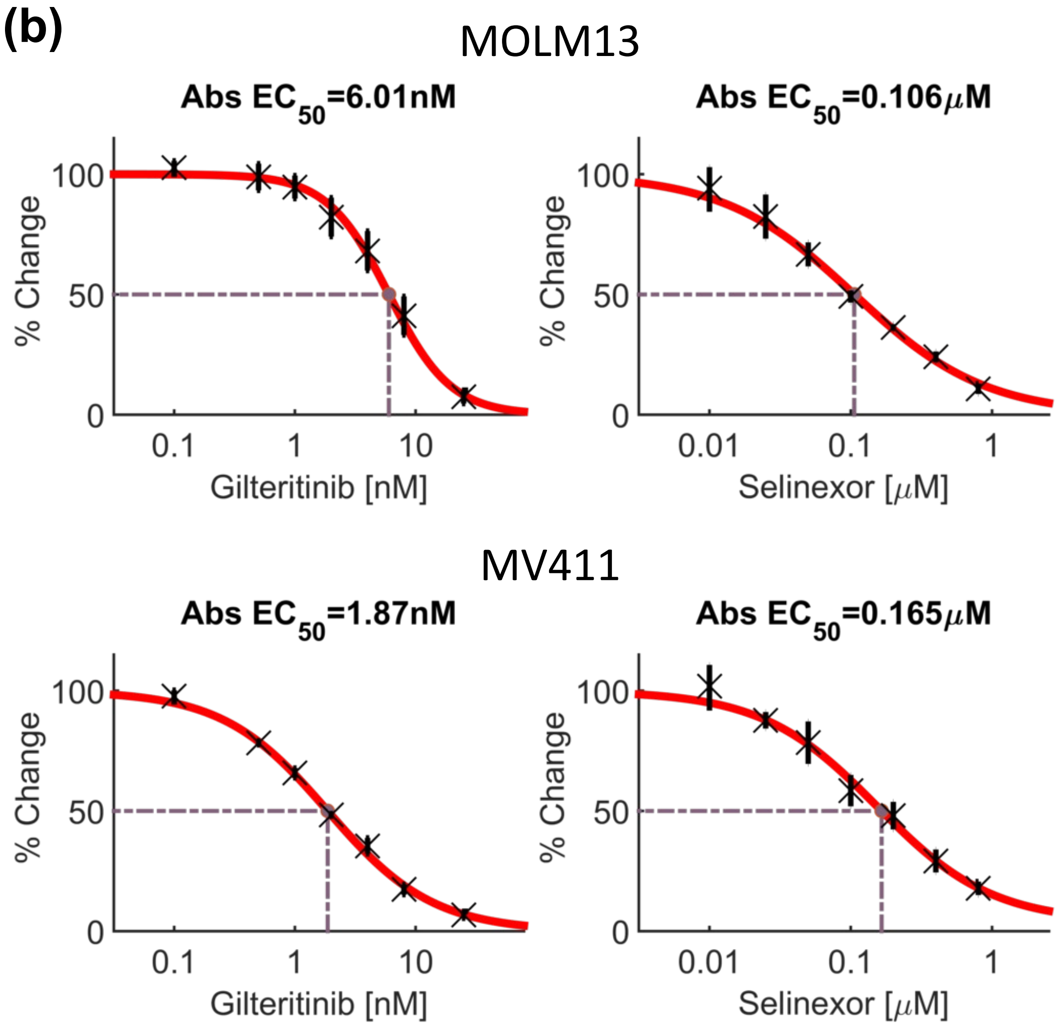

(c)

Synergy calculation derivation:

$$[\ln(\text{combination}) - \ln(\text{DMSO})] < [\ln(\text{FLT3 inhibitor}) - \ln(\text{DMSO})] + [\ln(\text{selinexor}) - \ln(\text{DMSO})]$$

$$\Rightarrow -[\ln(\text{combination}) - \ln(\text{DMSO})] > -[\ln(\text{FLT3 inhibitor}) - \ln(\text{DMSO})] - [\ln(\text{selinexor}) - \ln(\text{DMSO})]$$

$$\Rightarrow -\ln(\text{combination}) + \ln(\text{DMSO}) > -\ln(\text{FLT3 inhibitor}) + \ln(\text{DSMO}) - \ln(\text{selinexor}) + \ln(\text{DMSO})$$

$$\Rightarrow \ln(\text{DMSO}) - \ln(\text{combination}) + \ln(\text{FLT3 inhibitor}) - \ln(\text{DMSO}) + \ln(\text{selinexor}) - \ln(\text{DMSO}) > 0$$

$$\Rightarrow [\ln(\text{FLT3 inhibitor}) + \ln(\text{selinexor})] - [\ln(\text{combination}) + \ln(\text{DSMO})] > 0$$

$$\Rightarrow e^{[\ln(\text{FLT3 inhibitor}) + \ln(\text{selinexor})] - [\ln(\text{combination}) + \ln(\text{DSMO})]} > e^{0}$$

$$\Rightarrow [e^{\ln(\text{FLT3 inhibitor})} e^{\ln(\text{selinexor})}] / [e^{\ln(\text{combination})} e^{\ln(\text{DSMO})}] > 1$$

$$\Rightarrow [e^{\ln(\text{iFLT3 inhibitor})} / e^{\ln(\text{DSMO})}] \times [e^{\ln(\text{selinexor})} / e^{\ln(\text{combination})}] > 1$$

$$\Rightarrow (\text{FLT3 inhibitor} / \text{DMSO}) \times (\text{selinexor} / \text{combination}) > 1$$

$$\Rightarrow (\text{FLT3 inhibitor} / \text{DMSO}) / (\text{combination} / \text{selinexor}) > 1$$

Figure S3: Additional analyses of synergy from combination proliferation assays. Single-agent dose-response curves for each drug are shown for (a) midostaurin and (b) gilteritinib. (c) Derivation of equation used to test for mathematical synergy. Data was first log transformed and a linear mixed effects model applied. Synergy ratio of selinexor with midostaurin and gilteritinib for (d) MOLM13 and (e) MV411 cell lines with confidence interval and p-value; synergy ratios >1 are shaded blue with dark blue indicating p<.001 and light blue indicating .001<p<.05.

(d)

MOLM13 synergy calculations:

Midostaurin (nM)	Selinexor (nM)						
	10	20	50	100	200	500	1000
1	1.06 (1.03, 1.1) p=.001	1.11 (1.05, 1.17) p<.001	1.16 (1.11, 1.21) p<.001	1.18 (1.11, 1.25) p<.001	1.15 (1.05, 1.26) p=.005	1.01 (0.85, 1.21) p=.882	1.06 (0.93, 1.21) p=.347
3	1.13 (1.08, 1.18) p<.001	1.18 (1.12, 1.26) p<.001	1.27 (1.21, 1.34) p<.001	1.3 (1.23, 1.37) p<.001	1.2 (1.07, 1.34) p=.002	1.01 (0.85, 1.19) p=.933	0.92 (0.77, 1.11) p=.374
10	1.26 (1.19, 1.34) p<.001	1.42 (1.3, 1.54) p<.001	1.39 (1.27, 1.51) p<.001	1.38 (1.29, 1.49) p<.001	1.42 (1.22, 1.65) p<.001	0.99 (0.81, 1.21) p=.928	0.81 (0.68, 0.96) p=.014
20	1.31 (1.22, 1.4) p<.001	1.42 (1.31, 1.53) p<.001	1.37 (1.26, 1.49) p<.001	1.46 (1.37, 1.57) p<.001	1.57 (1.27, 1.93) p<.001	0.9 (0.73, 1.11) p=.314	0.59 (0.51, 0.69) p<.001
50	1.36 (1.23, 1.51) p<.001	1.3 (1.18, 1.43) p<.001	1.35 (1.21, 1.5) p<.001	1.61 (1.45, 1.8) p<.001	1.65 (1.36, 2) p<.001	0.71 (0.55, 0.92) p=.01	0.43 (0.37, 0.51) p<.001
100	1.37 (1.21, 1.55) p<.001	1.31 (1.13, 1.51) p=.001	1.28 (1.11, 1.48) p=.001	1.47 (1.28, 1.7) p<.001	1.13 (0.94, 1.37) p=.188	0.52 (0.4, 0.66) p<.001	0.25 (0.22, 0.29) p<.001
300	1.22 (1.07, 1.38) p=.003	1 (0.89, 1.12) p=.993	0.95 (0.87, 1.03) p=.224	0.78 (0.68, 0.89) p=.001	0.56 (0.47, 0.68) p<.001	0.22 (0.14, 0.34) p<.001	0.13 (0.11, 0.16) p<.001

Gilteritinib (nM)	Selinexor (nM)						
	10	25	50	100	200	400	800
0.1	1.02 (0.94, 1.12) p=.57	1.02 (0.95, 1.11) p=.54	1.01 (0.95, 1.09) p=.7	1.02 (0.98, 1.06) p=.326	1.03 (0.97, 1.09) p=.397	1.01 (0.92, 1.11) p=.872	0.88 (0.7, 1.09) p=.231
0.5	1.06 (0.98, 1.15) p=.153	1.07 (0.98, 1.18) p=.13	1.09 (1.01, 1.18) p=.024	1.08 (1.03, 1.13) p=.002	0.99 (0.94, 1.05) p=.756	0.97 (0.9, 1.05) p=.48	0.9 (0.78, 1.04) p=.154
1	1.13 (1.04, 1.22) p=.004	1.14 (1.04, 1.25) p=.006	1.16 (1.06, 1.27) p=.002	1.13 (1.07, 1.18) p<.001	1.05 (1, 1.12) p=.07	1.07 (0.99, 1.15) p=.087	0.97 (0.79, 1.17) p=.712
2	1.2 (1.09, 1.32) p=.001	1.16 (1.04, 1.3) p=.011	1.23 (1.11, 1.36) p<.001	1.23 (1.16, 1.31) p<.001	1.08 (1.01, 1.15) p=.023	1.1 (1, 1.2) p=.048	0.97 (0.83, 1.13) p=.695
4	1.23 (1.1, 1.37) p=.001	1.26 (1.12, 1.43) p=.001	1.32 (1.15, 1.5) p<.001	1.29 (1.22, 1.38) p<.001	1.1 (1.02, 1.19) p=.014	1.12 (1.03, 1.22) p=.009	0.85 (0.69, 1.05) p=.132
8	1.4 (1.2, 1.64) p<.001	1.37 (1.17, 1.61) p<.001	1.25 (1.07, 1.46) p=.005	1.16 (1.05, 1.28) p=.004	1.05 (0.94, 1.17) p=.36	1.07 (0.95, 1.2) p=.265	0.71 (0.58, 0.87) p=.002
25	1.56 (1.07, 2.27) p=.022	1.2 (0.85, 1.68) p=.294	1.17 (0.83, 1.63) p=.364	1.58 (1.15, 2.17) p=.006	1.16 (0.75, 1.79) p=.501	0.44 (0.33, 0.6) p<.001	0.24 (0.16, 0.37) p<.001

(e)

MV411 synergy calculations:

Midostaurin (nM)	Selinexor (nM)						
	10	20	50	100	200	500	1000
1	0.97 (0.91, 1.04) p=.35	0.95 (0.88, 1.02) p=.171	1.01 (0.93, 1.09) p=.809	1 (0.93, 1.07) p=.981	0.96 (0.84, 1.09) p=.517	0.94 (0.8, 1.11) p=.445	0.92 (0.76, 1.11) p=.36
3	1.02 (0.96, 1.09) p=.441	0.98 (0.91, 1.07) p=.687	1.06 (0.98, 1.15) p=.153	1.09 (0.99, 1.18) p=.064	1.06 (0.93, 1.2) p=.381	1 (0.82, 1.21) p=.961	0.84 (0.69, 1.01) p=.069
10	1.07 (0.99, 1.15) p=.069	1.1 (1, 1.21) p=.04	1.23 (1.13, 1.34) p<.001	1.18 (1.06, 1.3) p=.003	1.09 (0.92, 1.29) p=.302	0.84 (0.7, 1.02) p=.084	0.8 (0.62, 1.02) p=.068
20	1.17 (1.1, 1.26) p<.001	1.26 (1.13, 1.39) p<.001	1.37 (1.25, 1.51) p<.001	1.38 (1.24, 1.53) p<.001	1.11 (0.95, 1.29) p=.169	0.8 (0.65, 0.99) p=.037	0.67 (0.55, 0.83) p<.001
50	1.3 (1.22, 1.39) p<.001	1.38 (1.26, 1.52) p<.001	1.32 (1.21, 1.44) p<.001	1.27 (1.1, 1.47) p=.002	0.95 (0.82, 1.1) p=.47	0.66 (0.53, 0.81) p<.001	0.6 (0.49, 0.73) p<.001
100	1.4 (1.28, 1.53) p<.001	1.36 (1.21, 1.51) p<.001	1.25 (1.11, 1.4) p=.001	1.19 (1.02, 1.39) p=.025	0.79 (0.67, 0.92) p=.004	0.51 (0.4, 0.65) p<.001	0.37 (0.3, 0.45) p<.001
300	1.69 (1.53, 1.87) p<.001	1.78 (1.59, 2.01) p<.001	1.56 (1.39, 1.74) p<.001	1.04 (0.87, 1.25) p=.64	0.56 (0.44, 0.71) p<.001	0.36 (0.28, 0.46) p<.001	0.28 (0.22, 0.37) p<.001

Gilteritinib (nM)	Selinexor (nM)						
	10	25	50	100	200	400	800
0.1	1.04 (0.98, 1.11) p=.202	1.03 (0.98, 1.09) p=.218	1.04 (0.95, 1.13) p=.373	1 (0.91, 1.1) p=.991	0.98 (0.88, 1.09) p=.731	0.92 (0.82, 1.03) p=.157	0.88 (0.78, 0.99) p=.029
0.5	1.03 (0.95, 1.11) p=.492	1.02 (0.97, 1.08) p=.422	1.04 (0.96, 1.13) p=.311	1.03 (0.93, 1.14) p=.58	1 (0.91, 1.11) p=.935	0.88 (0.79, 0.99) p=.035	0.78 (0.7, 0.87) p<.001
1	1.15 (1.07, 1.23) p<.001	1.09 (1.02, 1.16) p=.011	1.1 (1.01, 1.2) p=.026	1.06 (0.97, 1.16) p=.17	1.01 (0.91, 1.12) p=.875	0.81 (0.72, 0.91) p=.001	0.69 (0.61, 0.77) p<.001
2	1.22 (1.11, 1.34) p<.001	1.17 (1.09, 1.26) p<.001	1.21 (1.09, 1.35) p=.001	1.11 (1, 1.22) p=.047	1 (0.89, 1.11) p=.928	0.74 (0.65, 0.84) p<.001	0.6 (0.53, 0.69) p<.001
4	1.32 (1.19, 1.47) p<.001	1.15 (1.02, 1.29) p=.025	1.17 (1.02, 1.35) p=.029	1.12 (0.99, 1.26) p=.062	0.96 (0.84, 1.09) p=.507	0.63 (0.54, 0.73) p<.001	0.5 (0.43, 0.59) p<.001
8	1.32 (1.13, 1.53) p=.001	1.09 (0.94, 1.27) p=.243	1.09 (0.89, 1.34) p=.39	1 (0.87, 1.15) p=.993	0.81 (0.69, 0.95) p=.013	0.53 (0.44, 0.63) p<.001	0.4 (0.34, 0.48) p<.001
25	1.65 (1.2, 2.25) p=.003	1.26 (0.93, 1.72) p=.133	1.21 (0.86, 1.69) p=.263	0.86 (0.64, 1.15) p=.289	0.53 (0.39, 0.71) p<.001	0.26 (0.2, 0.35) p<.001	0.19 (0.14, 0.27) p<.001

(a) Mutational status of primary samples used in MTS assays

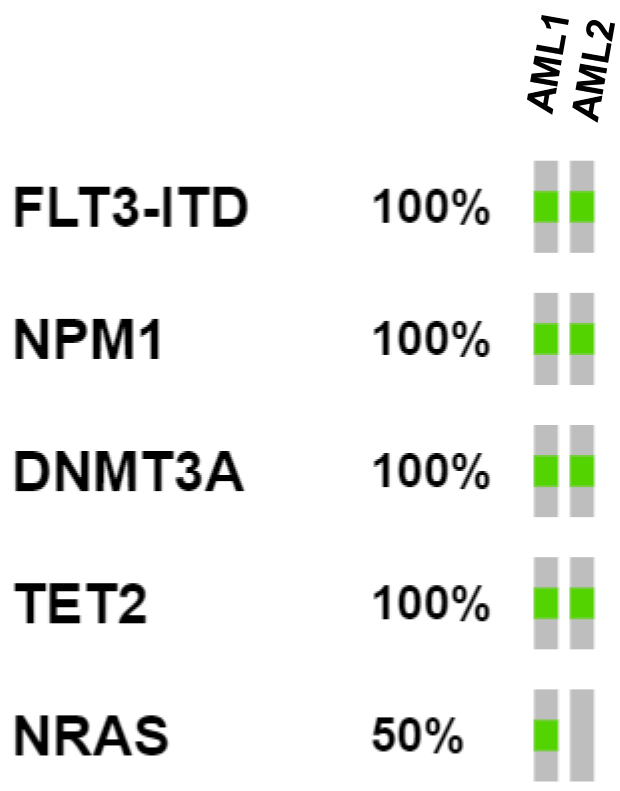

(b) AML1 primary patient synergy calculations:

Midostaurin (nM)	Selinexor (μM)				
	0.001	0.01	0.1	1	10
1	1.01<br>(0.93, 1.1)<br>0.762	0.94<br>(0.86, 1.04)<br>0.224	1.02<br>(0.79, 1.31)<br>0.883	1.07<br>(0.83, 1.38)<br>0.588	1.06<br>(0.86, 1.31)<br>0.588
10	0.83<br>(0.75, 0.93)<br>0.003	0.71<br>(0.62, 0.81)<br><0.001	0.94<br>(0.75, 1.19)<br>0.594	0.85<br>(0.68, 1.07)<br>0.163	0.9<br>(0.72, 1.11)<br>0.297
100	0.84<br>(0.76, 0.94)<br>0.003	0.71<br>(0.67, 0.76)<br><0.001	0.93<br>(0.74, 1.16)<br>0.495	0.65<br>(0.51, 0.81)<br>0.001	0.71<br>(0.61, 0.84)<br><0.001

Gilteritinib (nM)	Selinexor (μM)				
	0.001	0.01	0.1	1	10
1	0.96<br>(0.86, 1.07)<br>0.404	0.94<br>(0.86, 1.02)<br>0.127	0.95<br>(0.85, 1.07)<br>0.366	1.01<br>(0.91, 1.12)<br>0.795	0.92<br>(0.77, 1.1)<br>0.299
10	1.01<br>(0.88, 1.15)<br>0.918	0.92<br>(0.86, 0.97)<br>0.011	0.98<br>(0.86, 1.12)<br>0.757	0.98<br>(0.83, 1.16)<br>0.826	1.02<br>(0.86, 1.19)<br>0.834
100	1.05<br>(0.87, 1.28)<br>0.548	0.95<br>(0.8, 1.14)<br>0.543	0.87<br>(0.72, 1.05)<br>0.122	0.93<br>(0.71, 1.23)<br>0.576	0.71<br>(0.56, 0.89)<br>0.008
1000	1.35<br>(1.1, 1.66)<br>0.01	1.16<br>(0.98, 1.38)<br>0.077	0.81<br>(0.68, 0.98)<br>0.032	0.75<br>(0.61, 0.94)<br>0.018	0.39<br>(0.32, 0.48)<br><0.001
10000	1.4<br>(1.07, 1.83)<br>0.02	1.37<br>(1.04, 1.82)<br>0.031	0.89<br>(0.7, 1.14)<br>0.312	1.18<br>(0.58, 2.38)<br>0.608	0.79<br>(0.42, 1.51)<br>0.425

(c) AML2 primary patient synergy calculations:

Midostaurin (nM)	Selinexor (μM)				
	0.001	0.01	0.1	1	10
1	1.04<br>(0.93, 1.17)<br>0.469	0.91<br>(0.8, 1.02)<br>0.102	0.95<br>(0.82, 1.09)<br>0.418	1<br>(0.86, 1.17)<br>0.978	1<br>(0.85, 1.17)<br>0.961
10	1.06<br>(0.87, 1.3)<br>0.533	0.98<br>(0.75, 1.29)<br>0.905	0.91<br>(0.72, 1.15)<br>0.406	0.92<br>(0.72, 1.17)<br>0.465	0.91<br>(0.7, 1.18)<br>0.45
100	0.96<br>(0.84, 1.1)<br>0.569	0.83<br>(0.73, 0.94)<br>0.005	0.86<br>(0.74, 0.98)<br>0.03	0.77<br>(0.65, 0.91)<br>0.003	0.8<br>(0.67, 0.94)<br>0.011

Gilteritinib (nM)	Selinexor (μM)				
	0.001	0.01	0.1	1	10
1	0.99<br>(0.76, 1.27)<br>0.896	0.93<br>(0.72, 1.19)<br>0.497	1.08<br>(0.83, 1.42)<br>0.512	1.08<br>(0.84, 1.38)<br>0.515	1.41<br>(1.02, 1.96)<br>0.042
10	0.86<br>(0.72, 1.02)<br>0.075	0.93<br>(0.78, 1.1)<br>0.337	0.95<br>(0.79, 1.14)<br>0.53	0.97<br>(0.8, 1.18)<br>0.742	1.18<br>(0.83, 1.69)<br>0.315
100	0.71<br>(0.57, 0.89)<br>0.007	0.75<br>(0.59, 0.95)<br>0.022	0.78<br>(0.64, 0.96)<br>0.026	0.66<br>(0.53, 0.82)<br>0.003	1.23<br>(0.93, 1.62)<br>0.123
1000	0.9<br>(0.64, 1.26)<br>0.501	0.84<br>(0.58, 1.21)<br>0.307	0.68<br>(0.5, 0.92)<br>0.018	0.63<br>(0.47, 0.84)<br>0.006	0.66<br>(0.43, 0.99)<br>0.047
10000	1.12<br>(0.84, 1.5)<br>0.383	0.96<br>(0.59, 1.57)<br>0.865	0.64<br>(0.48, 0.86)<br>0.008	0.57<br>(0.41, 0.77)<br>0.003	0.32<br>(0.23, 0.47)<br><0.001

(d) Single-agent dose response of primary samples in MTS assays

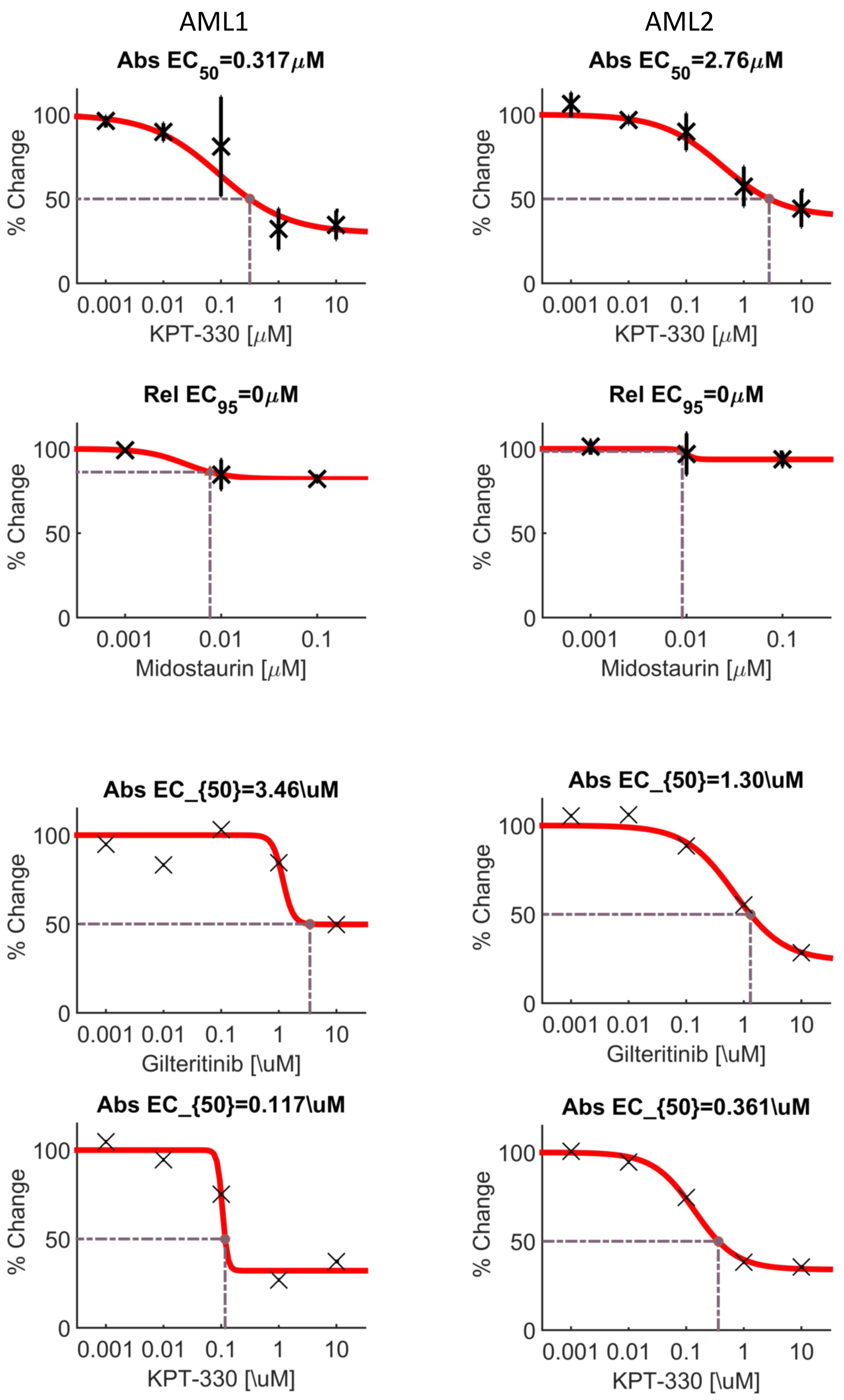

Figure S4: Primary samples used in MTS assays. (a) Mutations present in each sample. Synergy ratio of selinexor with midostaurin and gilteritinib was calculated as in Figure S3 for (b) AML1 primary patient sample and (c) AML2 primary patient sample with confidence interval and p-value; synergy ratios >1 and p<.05 are shaded with blue. This analysis was limited by fewer samples as compared to cell line experiments. (d) Single-agent dose-response curves of individual patient samples from combination MTS assays.

(a)

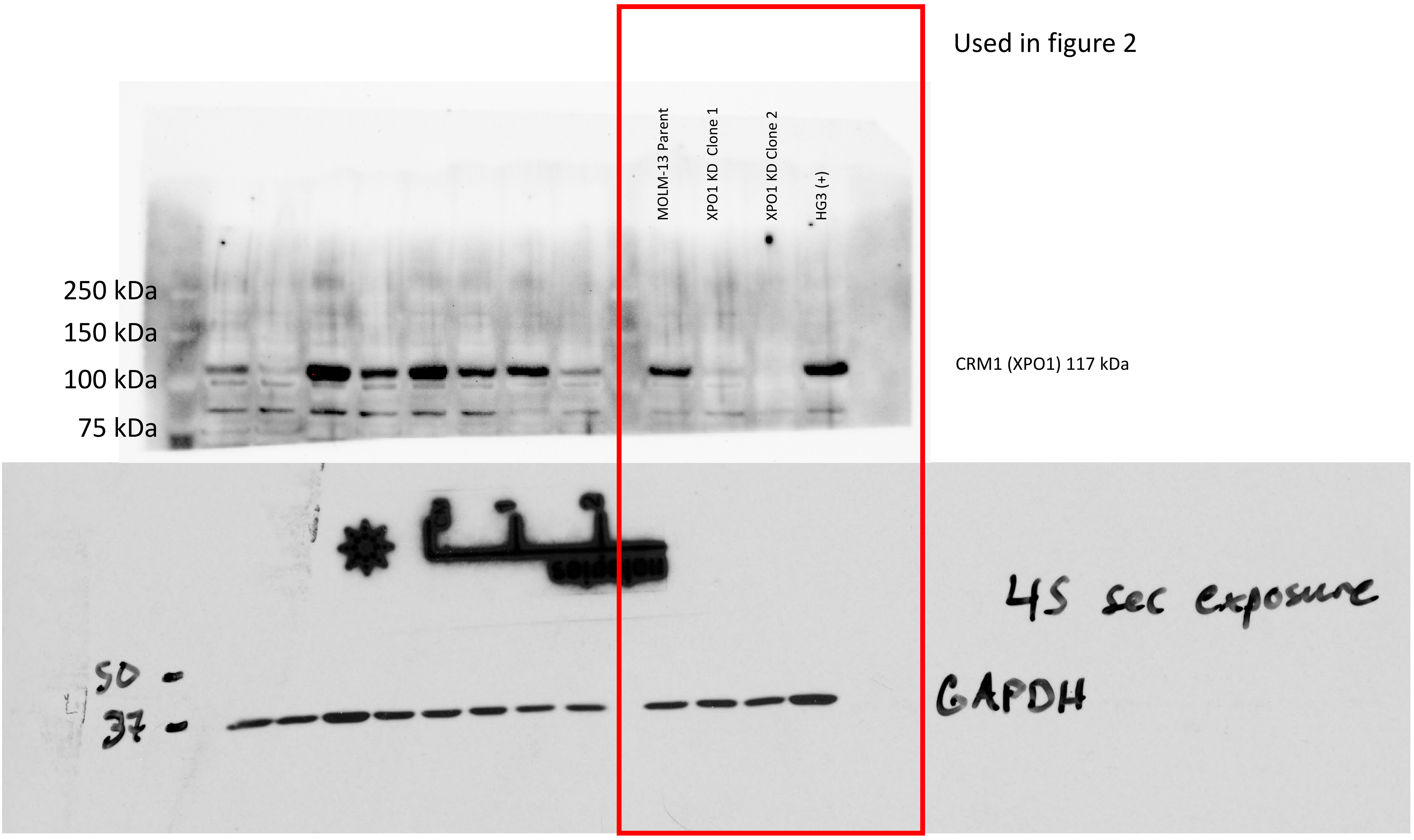

(b)

Lane No.	Sample	Adj. Total Band Vol. (Int)	Total Band Vol. (Int)	Adj. Total Lane Vol. (Int)	Total Lane Vol. (Int)	Bkgd. Vol. (Int)	Norm. Factor
1		17320002	44877200	23476876	138441934	114965058	1
2		5068410	26029233	15316815	104363967	89047152	0.848281089
3		165382448	205338518	175463134	290228862	114765728	0.682100763
4		33387264	55386279	42644820	128155050	85510230	0.831831271
5		91251504	122636430	100442625	197789379	97346754	1.010744594
6		35669532	56410281	44538024	130871790	86333766	1.117159992
7		51592419	75755794	59664733	152072877	92408144	1.282913767
8		11749656	34769784	16794086	106175034	89380948	1.418712725
9	MOLM-13 Parent	38770032	70588496	44601664	156370608	111768944	1.315632215
10	XPO1 KD Clone 1	5637429	28054533	9490917	83960166	74469249	1.090986156
11	XPO1 KD Clone 2	2556651	21646845	5901492	72929118	67027626	1.092529758
12	HG3 (+)	71961606	98697180	79268931	167635182	88366251	0.753128244

(c)

Sample	Anti-XPO1	Anti-GAPDH	Percent	Relative density
MOLM-13 Parent	12187.167	13107.125	92.98	1.00
XPO1 KD Clone 1	2457.861	13327.296	18.44	19.83
XPO1 KD Clone 2	1230.912	12480.296	9.86	10.60
HG3 (+ control)	18959.874	20675.024	91.70	98.62

Figure S5: Immunoblot analysis. (a) Uncropped scan of immunoblot from Figure 2. (b) Image Lab 6.0.1 analysis of anti-XPO1. (c) Densitometry analysis using ImageJ.
